# Influence of Tracheostomy Level on Surgical Management of Pediatric Airway Stenosis

**DOI:** 10.1002/lary.70569

**Published:** 2026-04-15

**Authors:** Alexandre Waldmeyer, Kishore Sandu, François Gorostidi

**Affiliations:** ^1^ Department of Otorhinolaryngology and Head and Neck Surgery Centre Hospitalier Universitaire Vaudois (CHUV) Lausanne Switzerland

**Keywords:** cricotracheal resection, laryngotracheal reconstruction, laryngotracheal stenosis, pediatric tracheostomy, subglottic stenosis

## Abstract

**Objectives:**

To assess whether pre‐existing tracheostomy location influences outcomes of open airway surgery for pediatric laryngotracheal stenosis (LTS) and to propose a pragmatic framework for tracheostomy placement.

**Methods:**

A retrospective review included 104 tracheostomized children who underwent open airway surgery for LTS between 1978 and 2022. Collected variables included stenosis characteristics, comorbidities, tracheostomy location, surgical technique, decannulation outcomes, complications, and revision surgery. Tracheostomy location was independently reviewed by two experienced pediatric airway surgeons and categorized as optimal, suboptimal, or nonoptimal based on stenosis characteristics and patient comorbidities. Surgical outcomes were compared across groups.

**Results:**

Most suboptimal tracheostomies were in a classic position (60/62, 97%). In cricotracheal resection, the suboptimal group required a greater extent of resection (median 5 rings [IQR 4–5]) compared with the optimal (4 rings [3–4], *p* = 0.027) and nonoptimal groups (3 rings [2.5–4], *p* = 0.004). Among double‐stage procedures, time to decannulation was longest in nonoptimal patients (median 11.7 months [IQR 4.6–19.8]) compared with suboptimal (4 months [2.5–8.6], *p* = 0.032) and optimal groups (3.8 months [2–4.9], *p* = 0.027). Tracheostomy relocation was less frequent in the optimal group (29% vs. 57%–67% in the other groups). Tracheostomy‐related complications and revision procedures were more frequent in the suboptimal group.

**Conclusions:**

Preoperative tracheostomy location influences surgical complexity (single‐stage vs. double‐stage procedure), resection length, need for tracheostomy relocation, complication rates, and time to decannulation in pediatric LTS surgery. Planning tracheostomy location with the anticipated reconstructive pathway in mind may reduce morbidity and improve outcomes.

**Level of Evidence:**

3.

## Graphical abstract

Tracheostomy location is a critical determinant of surgical complexity and outcomes in pediatric laryngotracheal stenosis. Misplacement may lead to longer tracheal resections, an increased need for stoma relocation, more complex reconstruction, and secondary stenosis. Strategic planning of tracheostomy position can improve surgical outcomes.

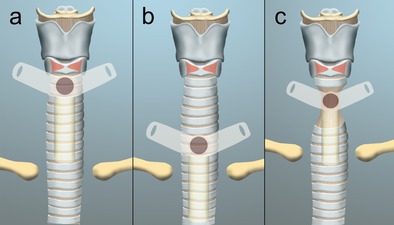

## 1 Introduction

Tracheostomy remains a cornerstone in the management of laryngotracheal stenosis (LTS) in children and can significantly affect family quality of life. It disrupts developmental functions such as speech, increases healthcare costs, and requires parental training for safe home care. Children are particularly vulnerable to tracheostomy‐related complications, including infections, mucus plugging, accidental decannulation, and iatrogenic stenosis due to cannula‐related trauma [1]. Mucus plugging and accidental decannulation are associated with a reported mortality risk of 1%–2% [2]. When tracheostomy is required, decannulation remains a major goal in pediatric LTS treatment [1, 2, 3, 4, 5].

LTS is a congenital, acquired, or mixed narrowing of the laryngotracheal airway, and can involve an isolated subsite (supraglottic, glottic, subglottic, or tracheal) or multiple sites [6, 7, 8, 9]. Symptoms range from mild stridor to life‐threatening obstruction [6, 10]. Most acquired cases (≈90%) result from endotracheal intubation, especially in children younger than 5 years, whose narrow airway (particularly between the cricoid and first tracheal ring) is more vulnerable to mucosal injury and fibrosis [6, 9, 11, 12]. Subglottic stenosis (SGS) is defined as an airway diameter < 4.0 mm in full‐term or < 3.0 mm in preterm neonates. It is congenital in 5%–6% of cases, acquired in 55%–95%, and mixed in 0%–39% [2, 6, 7, 8, 9, 13]. The incidence of post‐intubation SGS in children ranges from 0% to 2% [7]. Other acquired causes include infections, trauma, neoplasms, and autoimmune diseases [2, 13]. Prognosis is influenced by stenosis cause, severity, extent, and comorbidities or syndromic conditions (e.g., DiGeorge syndrome) [10]. Severity is assessed with the Cotton–Myer scale for SGS, the Bogdasarian classification for posterior glottic stenosis (GS), and the Cohen classification for laryngeal webs (LW). LTS remains challenging to categorize, treat, and study due to its heterogeneous etiology, presentation, and comorbidities [3, 6].

Definitive treatment for LTS is surgical. Endoscopic approaches are appropriate for mild disease, while open procedures are indicated for severe and complex stenoses. In our institution, laryngotracheal reconstruction (LTR), using costal cartilage grafts to expand the airway, is favored in selected glottic or low‐grade SGS. Cricotracheal resection (CTR), involving resection of the stenotic segment and an end‐to‐end anastomosis, is preferred for high‐grade SGS. An extended CTR (eCTR) combines CTR with posterior glottic expansion using a posterior rib graft [4, 6, 10, 13, 14]. These procedures may be performed in single‐stage (SS) (airway surgery to treat the stenosis and concomitantly remove the tracheostomy) or double‐stage (DS) fashion (treat the LTS first, then remove the tracheostomy) [4, 14]. Success of these operations to achieve decannulation is around 95%–100% for isolated SGS, but drops to 68%–90% in patients with comorbidities, synchronous airway anomalies, or multilevel stenoses [6, 10, 13, 14, 15].

In patients with LTS and airway compromise, tracheostomy is often required. In emergency settings, the tracheostomy might be performed by non‐airway surgeons, sometimes without anticipating the future airway reconstructive strategy. As a result, tracheostomy site selection may significantly influence subsequent management, including surgical technique (SS vs. DS), outcomes, and decannulation results [6, 13]. In this report, we reviewed a 44‐year experience from a single quaternary referral center to assess the impact of tracheostomy position in pediatric LTS and propose a pragmatic framework for tracheostomy placement with future airway reconstruction in mind.

## 2 Materials and Methods

### 2.1 Study Objectives

- *Primary objective*: To assess whether the location of pre‐existing tracheostomy influences outcomes of open reconstructive surgery for pediatric LTS.
- *Secondary objectives*:
  1. Quantify non‐diseased healthy trachea tissue resected to treat the LTS in CTR and achieve decannulation.
  2. Propose a pragmatic framework to guide tracheostomy placement based on stenosis characteristics and patient condition.

### 2.2 Inclusion Criteria

All tracheostomized pediatric patients (< 18 years old) who underwent open reconstructive surgery for LTS at our institution between March 1, 1978, and June 30, 2022, were included. Collected data comprised demographics, comorbidities, stenosis characteristics, tracheostomy location, surgical details, complications, and decannulation outcomes.

### 2.3 Exclusion Criteria

- Tracheostomy unrelated to LTS.
- Parents' refusal to allow data use.
- Missing key information.

### 2.4 Data Collection

After Ethics Committee approval (CER‐VD 2022‐02197), data were extracted from all available medical records. A total of 104 patients were included. LTS were classified by localization and graded using the Bogdasarian classification for GS, the Cohen classification for LW, and the Cotton–Myer scale for isolated SGS, as well as for multisite stenoses (supraglottic‐glottic stenosis [Sp‐GS], glotto‐subglottic stenosis [G‐SGS], subglotto‐tracheal stenosis [SG‐TS], glotto‐subglotto‐tracheal stenosis [G‐SG‐TS], tracheal stenosis [TS]). All classifications were based on preoperative endoscopy.

The tracheostomy location was classified as high when placed between the cricoid cartilage and first or first and second tracheal ring, classic between second and third or third and fourth tracheal ring, low between fourth and fifth or fifth and sixth tracheal ring, and very low between sixth and seventh tracheal ring or below.

### 2.5 Tracheostomy Position Classification

All pretreatment data were independently reviewed by two senior pediatric airway surgeons (F.G. and K.S.). Tracheostomy location was classified as optimal, suboptimal, or nonoptimal a priori, based solely on preoperative information, including stenosis site and extent (isolated vs. multilevel), stenosis severity, tracheostomy level, and patient comorbidities. F.G. and K.S. based their classification on the anticipated reconstructive strategy at our institution (LTR vs. CTR; SS vs. DS) and were blinded to the definitive surgical approach and postoperative outcomes at the time of classification.

In our institution, tracheal involvement is measured by the number of tracheal rings rather than absolute distance, as the tracheal ring height varies with age.

Two surgical groups were defined: LTR and CTR, our preference being LTR in low‐grade stenosis (selected advanced Grade I, Grade II, and Low‐Grade III) and CTR in high‐grade cases (High Grade III and IV). For SS procedures, a high tracheostomy was considered optimal, allowing excision of the stoma with the stenosis during SS‐CTR and minimizing healthy tissue resection. A SS‐LTR permits stoma closure with anterior costal cartilage graft. For DS surgery, low or very low tracheostomies were deemed optimal, avoiding further downward displacement (relocation) and preserving healthy trachea.

Tracheostomy positioning was considered optimal when aligned with expert recommendations based on stenosis characteristics, comorbidities, and anticipated reconstructive strategy. Suboptimal positioning indicated partial discordance, while nonoptimal reflected clear discordance, with the tracheostomy placed opposite to the recommended level (e.g., high instead of low/very low, or vice versa) (Table S1).

### 2.6 Complications

Only tracheostomy‐related complications requiring medical or surgical treatment (endoscopic or open revision) were considered (e.g., anastomotic dehiscence, suprastomal collapse, restenosis at the tracheostomy site (such as A‐shape deformity), cartilage graft infection due to stoma proximity, tracheostoma infection, and tracheocutaneous fistula). Granulation tissue without airway obstruction was excluded.

### 2.7 Data Analysis

Because of the rarity of the condition and small heterogeneous subgroups, no a priori power calculation was performed, and analyses were considered exploratory. Descriptive statistics were used. Normality was assessed using the Shapiro–Wilk test; as at least one subgroup violated assumptions, all quantitative variables were reported as median (IQR), and between‐group differences were assessed using nonparametric tests (Kruskal–Wallis and Mann–Whitney *U*). Effect size was expressed using the Hodges–Lehmann estimator with 95% confidence intervals. Categorical variables were compared using Fisher's exact test. A *p* value < 0.05 was considered significant. Analyses were performed in Stata/SE 18 and Microsoft Excel.

## 3 Results

### 3.1 Preoperative Cohort Characteristics

Of the 104 patients, 24 (23%) had an optimal, 62 (60%) a suboptimal, and 18 (17%) a nonoptimal tracheostomy. The main stenosis sites were SGS in 61 patients (59%), G‐SGS in 32 (31%), and GS in 5 (5%). Most patients had high‐grade stenosis: 56 (54%) Cotton–Myer Grade III and 20 (19%) Grade IV. Three patients (3%) had a Bogdasarian Grade III stenosis and two (2%) a Grade IV. Comorbidities were present in 78 (75%) patients (categories were non‐mutually exclusive: 41% ENT, 32% pulmonary, 25% gastrointestinal) and prematurity in 53 (51%) patients, with 60% in suboptimal, 33% in optimal, and 44% in nonoptimal groups. Previous airway interventions were reported in 37 patients (36%), consisting of endoscopic procedures such as laser treatment in 16 (15%) and dilations in 15 (14%). Previous open surgical procedures were also present, with 11 patients (11%) having undergone a LTR and 3 patients (3%) a CTR (Table 1).

**TABLE 1:** Study cohort characteristics.

Characteristics	All *n* = 104, *N* (%), median (IQR)	Optimal *n* = 24, *N* (%), median (IQR)	Suboptimal *n* = 62, *N* (%), median (IQR)	Nonoptimal *n* = 18, *N* (%), median (IQR)	*p*
*Preoperative*					
Sex					
Male	58 (56%)	14 (58%)	33 (53%)	11 (61%)	
Female	46 (44%)	10 (42%)	29 (47%)	7 (39%)	
Origin					
Switzerland	14 (13%)	4 (17%)	6 (10%)	4 (22%)	
Europe	81 (78%)	17 (71%)	52 (84%)	11 (61%)	
Others	9 (9%)	3 (12%)	4 (6%)	3 (17%)	
Stenosis location					
Sp‐GS	1 (1%)	0	0	1 (6%)	
GS	5 (5%)	3 (13%)	2 (3%)	0	
G‐SGS	32 (31%)	6 (25%)	21 (34%)	5 (28%)	
SGS	61 (59%)	13 (54%)	36 (58%)	12 (67%)	
SG‐TS	1 (1%)	0	1 (2%)	0	
TS^a^	1 (1%)	0	1 (2%)	0	
G‐SG‐TS	1 (1%)	1 (4%)	0	0	
LW	2 (2%)	1 (4%)	1 (2%)	0	
Stenosis grade					
CM I	7 (7%)	0	6 (10%)	1 (6%)	
CM II	14 (13%)	1 (4%)	11 (18%)	2 (11%)	
CM III	56 (54%)	13 (54%)	32 (52%)	11 (61%)	
CM IV	20 (19%)	6 (25%)	10 (16%)	4 (22%)	
Bgd III	3 (3%)	1 (4%)	2 (3%)	0	
Bgd IV	2 (2%)	2 (8%)	0	0	
Cn IV	2 (2%)	1 (4%)	1 (2%)	0	
Etiology					
Acquired	71 (68%)	15 (63%)	41 (66%)	15 (83%)	
Congenital	18 (17%)	6 (25%)	9 (15%)	3 (17%)	
Mixt	15 (14%)	3 (13%)	12 (19%)	0	
Comorbidities^b^					
Yes	78 (75%)	20 (83%)	49 (79%)	9 (50%)	
No	26 (25%)	4 (17%)	13 (21%)	9 (50%)	
Cardiac	23 (22%)	7 (29%)	7 (11%)	1 (6%)	
Pulmonary	33 (32%)	8 (33%)	22 (35%)	3 (17%)	
Gastrointestinal	26 (25%)	5 (21%)	18 (29%)	3 (17%)	
Neurologic	11 (11%)	4 (17%)	6 (10%)	1 (6%)	
ENT	43 (41%)	10 (42%)	28 (45%)	5 (28%)	
Others	14 (13%)	7 (29%)	9 (15%)	0	
Prematurity					
Yes	53 (51%)	8 (33%)	37 (60%)	8 (44%)	
No	51 (49%)	16 (67%)	25 (40%)	10 (56%)	
Previous treatments					
Yes	37 (36%)	9 (37%)	20 (32%)	8 (44%)	
No	67 (64%)	15 (63%)	42 (68%)	10 (56%)	
Laser	16 (15%)	2 (8%)	7 (11%)	7 (39%)	
Dilations	15 (14%)	4 (17%)	7 (11%)	4 (22%)	
LTR	11 (11%)	2 (8%)	7 (11%)	2 (11%)	
PCTR	3 (3%)	1 (4%)	2 (3%)	0	
Stent	4 (4%)	0	3 (5%)	1 (6%)	
Laryngofissure	1 (%)	0	1 (2%)	0	
Total number of previous treatments^c^					
Laser	35	3	17	15	
Dilations	16	4	9	3	
LTR	15	2	11	2	
PCTR	3	1	2	0	
Stent	5	0	4	1	
Laryngofissure	1	0	1	0	
Tracheostomy location					
High	16 (15%)	2 (8%)	2 (3%)	12 (67%)	
Classic	61 (59%)	1 (4%)	60 (97%)	0	
Low	23 (22%)	19 (79%)	0	4 (22%)	
Very low	4 (4%)	2 (8%)	0	2 (11%)	
*Operative and postoperative*					
Age at operation (years)	2.5 (1.5–5.3)	2.3 (1.3–4.8)	2.6 (1.6–4.4)	3.6 (1.1–7.5)	0.043^g^
LTR	26 (25%)	4 (17%)	18 (29%)	4 (22%)	
SS	4 (4%)	1 (4%)	2 (3%)	1 (5%)	
DS	22 (21%)	3 (13%)	16 (26%)	3 (17%)	
CTR	78 (75%)	20 (83%)	44 (71%)	14 (78%)	
SS	42 (40%)	9 (37%)	25 (40%)	8 (44%)	
DS	36 (35%)	11 (46%)	19 (31%)	6 (33%)	
Tracheal rings resected^d^	4 (3–5) (*n* = 71)	4 (3–4) (*n* = 17)	5 (4–5) (*n* = 42)	3 (2.5–4) (*n* = 12)	0.007^g^
Tracheostomy relocation	30 (52%) (*n* = 58)	4 (29%) (*n* = 14)	20 (57%) (*n* = 35)	6 (67%) (*n* = 9)	0.335^h^
Decannulation rate	91 (88%)	21 (88%)	54 (87%)	16 (89%)	1.000^h^
Time to decannulation (months)	0 (0–4.6)	0.8 (0–3.8)	0.9 (0–4)	0 (0–9.1)	0.917^g^
Decannulation rate in DS operations	45 (78%) (*n* = 58)	11 (79%) (*n* = 14)	27 (77%) (*n* = 35)	7 (78%) (*n* = 9)	1.000^h^
Time to decannulation in DS operations (months)	4.6 (2.7–8.6) (*n* = 58)	3.8 (2–4.9) (*n* = 14)	4 (2.5–8.6) (*n* = 35)	11.7 (4.6–19.8) (*n* = 9)	0.042^g^
Complications related to tracheostomy	31 (30%)	6 (25%)	20 (32%)	5 (28%)	0.833^h^
Endoscopic reinterventions^e^	24 (23%)	4 (17%)	16 (26%)	4 (22%)	0.829^h^
Open surgery reinterventions^e^	21 (20%)	4 (17%)	14 (23%)	3 (17%)	1.000^h^
Follow‐up (years)^f^	1 (0.3–3.5)	1.3 (0.3–2.2)	1.2 (0.4–3.7)	0.6 (0.3–3.5)	0.043^g^

*Note:* Percentages refer to patients.

Abbreviations: Bgd, Bogdasarian; CM, Cotton–Myer; Cn, Cohen; CTR, cricotracheal resection; DS, double‐stage; GS, glottic stenosis; G‐SGS, glotto‐subglottic stenosis; GS‐SG‐TS, glotto‐subglotto‐tracheal stenosis; LTR, laryngotracheal reconstruction; LW, laryngeal web; PCTR, partial cricotracheal resection; SGS, subglottic stenosis; SG‐TS, subglotto‐tracheal stenosi; Sp‐GS, supraglottic‐glottic stenosis; SS, single‐stage; TS, tracheal stenosis.

^a^ The patient presented with a stenosis reaching close to the cricoid and spanning 3–4 tracheal rings, for which a SS PCTR was performed.

^b^ Percentages are non‐mutually exclusive.

^c^ Procedure counts represent total number of interventions performed prior to definitive open airway reconstruction.

^d^ Seven patients underwent only partial cricoid resection without any tracheal ring resection.

^e^ Only patients with tracheostomy‐related complications.

^f^ No patient died during this period.

^g^ Performed with Kruskal–Wallis test.

^h^ Performed with Fisher's exact test.

### 3.2 Tracheostomy Characteristics

Tracheostomy locations were classified as classic in 61 (59%), low in 23 (22%), high in 16 (15%), and very low in 4 (4%).

**FIGURE 1:**
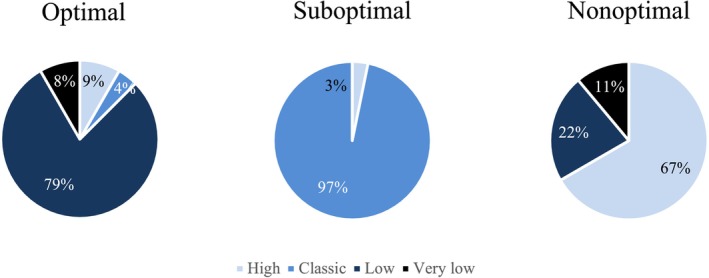
Distribution of tracheostomy levels (high/classic/low/very low) across optimal, suboptimal, and nonoptimal groups.

Optimal cases commonly had a low tracheostomy (19/24, 79%), suboptimal cases a classic tracheostomy (60/62, 97%), and nonoptimal cases a high tracheostomy (12/18, 67%) (Figure 1 and Table 1).

### 3.3 Operative and Postoperative Data

The median age at surgery was 2.5 years (IQR 1.5–5.3). Of the 104 patients, 78 (75%) underwent a CTR, including 42 (40%) SS and 36 (35%) DS procedures. LTR was performed in 26 patients (25%), comprising 4 (4%) SS and 22 (21%) DS (Table 1).

### 3.4 Tracheal Rings Resection

**FIGURE 2:**
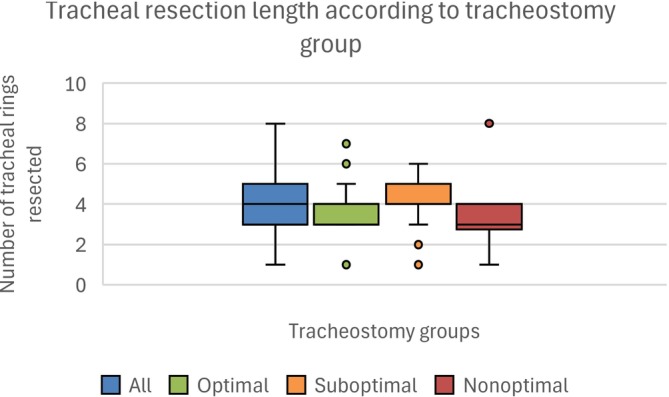
Tracheal resection length (number of rings) among patients undergoing cricotracheal resection with ≥ 1 ring resected, according to tracheostomy group (optimal, suboptimal, and nonoptimal). Box‐and‐whisker plot showing median and interquartile range. Patients with partial cricoid resection only (*n* = 7) were excluded.

In patients undergoing a CTR, 7 underwent partial cricoid resection only and 71 had resection of 1 or more tracheal rings. The number of tracheal rings resected was higher in the suboptimal group (median 5 [IQR 4–5]) compared with the optimal (4 [3–4], *p* = 0.027) and nonoptimal groups (3 [2.5–4], *p* = 0.004) (Figure 2, Table 1, and Table S2).

Similar patterns were observed in both SS and DS CTR procedures: in SS CTR, the suboptimal group had a higher number of rings resected (median 5 [IQR 4–5]) than the optimal (4 [3–4]) and nonoptimal subgroups (2.5 [2–4]); in DS CTR, the suboptimal subgroup also had greater resection (median 4 [IQR 4–5]) compared with the optimal (3 [2–4.5]) and nonoptimal subgroups (3 [3–4]) (Table 2).

**TABLE 2:** LTR operative and postoperative characteristics.

Characteristics	SS LTR				DS LTR			
	All *n* = 4, *N* (%)	Optimal *n* = 1or *N* (%)	Suboptimal *n* = 2 or *N* (%)	Nonoptimal *n* = 1 or *N* (%)	All *n* = 22, *N* (%), median (IQR)	Optimal *n* = 3, *N* (%), median (IQR)	Suboptimal *n* = 16, *N* (%), median (IQR)	Nonoptimal *n* = 3, *N* (%), median (IQR)
Tracheostomy relocation	NA	NA	NA	NA	6 (27%)	0	5 (31%)	1 (33%)
Decannulation rate	NA	NA	NA	NA	17 (77%)	3 (100%)	12 (75%)	2 (67%)
Time to decannulation (months)	NA	NA	NA	NA	2.9 (2.5–4.6)	3.8 (2.6–5.6)	2.8 (2.1–3.6)	8.7 (4.6–12.7)
Complications related to tracheostomy	1 (25%)	0	1 (50%)	0	5 (23%)	0	4 (25%)	1 (33%)
Endoscopic reinterventions	1 (25%)	0	1 (50%)	0	5 (23%)	0	4 (25%)	1 (33%)
Open surgery reinterventions	1 (25%)	0	1 (50%)	0	4 (18%)	0	3 (19%)	1 (33%)

*Note:* Subgroup analyses are descriptive due to small sample sizes.

Abbreviations: CTR, cricotracheal resection; DS, double‐stage; LTR, laryngotracheal reconstruction; SS, single‐stage.

### 3.5 Tracheostomy Relocation

Distal tracheostomy relocation was required in 30/58 DS patients (52%) and occurred respectively in 29%, 57%, and 67% in optimal, suboptimal, and nonoptimal subgroups (Table 1).

### 3.6 Decannulation Rate and Timing

**FIGURE 3:**
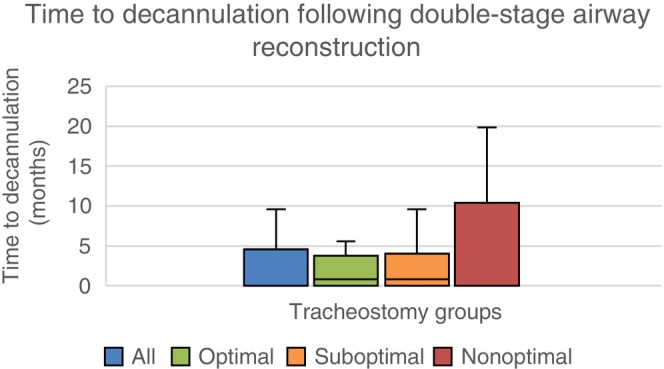
Time to decannulation (months) following double‐stage procedures, according to tracheostomy group. Box‐and‐whisker plot showing median and interquartile range.

Overall decannulation rate was 88% (91/104) and similar across groups (optimal 88%, suboptimal 87%, nonoptimal 89%). Among the 58 DS patients, 45 (78%) were decannulated, with comparable rates across groups, but time to decannulation differed and was longer in nonoptimal patients (median 11.7 [IQR 4.6–19.8]) compared to optimal (3.8 [IQR 2–4.9], *p* = 0.027) and suboptimal (4 [2.5–8.6], *p* = 0.032) (Table 1, Table S2, and Figure 3).

In DS LTR subgroup, decannulation rates varied across groups (100%, 75%, and 67% in the optimal, suboptimal, and nonoptimal groups, respectively), as did time to decannulation (median 3.8 months [IQR 2.6–5.6], 2.8 [2.1–3.6], and 8.7 [4.6–12.7], respectively) (Table 3).

**TABLE 3:** CTR operative and postoperative characteristics.

Characteristics	SS CTR				DS CTR			
	All *n* = 42, *N* (%), median (IQR)	Optimal *n* = 9, *N* (%), median (IQR)	Suboptimal *n* = 25, *N* (%), median (IQR)	Nonoptimal *n* = 8, *N* (%), median (IQR)	All *n* = 36, *N* (%), median (IQR)	Optimal *n* = 11, *N* (%), median (IQR)	Suboptimal *n* = 19, *N* (%), median (IQR)	Nonoptimal *n* = 6, *N* (%), median (IQR)
Tracheal rings resected^a^	4 (3–5) (*n* = 39)	4 (3–4) (*n* = 9)	5 (4–5) (*n* = 24)	2.5 (2–4) (*n* = 6)	4 (3–5) (*n* = 32)	3 (2–4.5) (*n* = 8)	4 (4–5) (*n* = 18)	3 (3–4) (*n* = 6)
Tracheostomy relocation	NA	NA	NA	NA	24 (67%)	4 (36%)	15 (79%)	5 (83%)
Decannulation rate	NA	NA	NA	NA	28 (78%)	8 (73%)	15 (79%)	5 (83%)
Time to decannulation (months)	NA	NA	NA	NA	5.5 (3.5–13.8)	3.8 (1.4–4.9)	7.5 (3.7–15.9)	11.7 (6.6–19.8)
Complications related to tracheostomy	9 (21%)	1 (11%)	7 (28%)	1 (12%)	16 (44%)	5 (45%)	8 (42%)	3 (50%)
Endoscopic reinterventions	6 (14%)	1 (11%)	5 (20%)	0	13 (36%)	3 (27%)	7 (37%)	3 (50%)
Open surgery reinterventions	7 (17%)	0	6 (24%)	1 (12%)	10 (28%)	4 (36%)	5 (26%)	1 (17%)

*Note:* Subgroup analyses are descriptive due to small sample sizes.

Abbreviations: CTR: cricotracheal resection; DS, double‐stage; LTR, laryngotracheal reconstruction; SS, single‐stage.

^a^ Seven patients underwent only partial cricoid resection without any tracheal ring resection.

Conversely, in the DS CTR subgroup, decannulation rate was slightly lower in the optimal group (73%) compared with the suboptimal and nonoptimal groups (79% and 83%, respectively), while median time to decannulation increased progressively (median 3.8 months [IQR 1.4–4.9], 7.5 [3.7–15.9], and 11.7 [6.6–19.8] in optimal, suboptimal, and nonoptimal groups, respectively) (Table 2).

### 3.7 Complications Related to Tracheostomy

Tracheostomy‐related complications occurred in 31 patients (30%) and were observed in 6 of 24 (25%) optimal, in 20 of 62 (32%) suboptimal, and in 5 of 18 (28%) nonoptimal patients (Table 1).

### 3.8 Revision Interventions

All patients with tracheostomy‐related complications required revision, either endoscopic, open surgery, or both. Endoscopic reintervention occurred in 4 (17%) optimal, 16 (26%) suboptimal, and 4 (22%) nonoptimal patients. Open surgery revision occurred in 4 (17%) optimal, 14 (23%) suboptimal, and 3 (17%) nonoptimal patients (Table 1).

## 4 Discussion

To our knowledge, this is the first study to evaluate the impact of the tracheostomy location on the surgical management of pediatric LTS. Tracheostomized patients referred for open airway reconstruction were classified into three categories—optimal, suboptimal, and nonoptimal—based on the relationship between tracheostomy location and the stenotic segment.

### 4.1 Influence of Tracheostomy Site on Open Airway Surgery Outcomes

#### 4.1.1 Influence of Suboptimal Tracheostomy on Tracheal Resection Length

Suboptimal tracheostomies, almost exclusively in the *classic position*, were associated with longer resections during CTR, requiring one to two additional healthy tracheal rings resected compared to the other groups. This can be explained by the overlap between the stoma and the planned anastomotic site, which necessitates excision of intervening healthy trachea to achieve a safe reconstruction. In addition, distal tracheostomy relocation is often required during DS‐CTR.

Available data suggest that up to 30% of the trachea may be safely resected in selected pediatric cases [16].

Reported complication rates after open LTS surgery range from 5% to 52% in the literature, with revision endoscopy and revision open surgery required in up to 50% and 22%–30% of cases, respectively [8, 16, 17].

When performing tracheostomy in case of incipient stenosis that may require CTR in the future, preservation of as much healthy trachea as possible is crucial, as excessive resection increases anastomotic tension and the risk of dehiscence [3, 14, 15, 18, 19]. Similar observations were reported by Hathiram et al., who demonstrated longer resections when the tracheostomy was placed fewer than four rings below the stenosis, resulting in stoma and anastomosis overlap [18]. In our study, tracheostomy‐related complications were more frequent in the suboptimal group (32%).

Most patients had a *classic* tracheostomy, reflecting standard textbook practice and limited anticipation of future reconstruction. Performing a tracheostomy in children is technically more challenging than in adults, as pediatric patients have a short and fatty neck, a small and pliable trachea, and less reliable surface landmarks; in addition, the hyoid bone may project over the thyroid cartilage [1, 20]. Premature infants were overrepresented in the suboptimal group, likely reflecting emergency indications, particularly small airway dimensions, and technical constraints at the time of tracheostomy placement. Pradeep et al. similarly reported the impact of emergency tracheostomy placement on subsequent resection length, with relatively distal placement from the stenosis [21].

#### 4.1.2 Influence of Nonoptimal Tracheostomy on Tracheostomy Relocation and Time to Decannulation

Nonoptimal tracheostomies, most commonly located in a *high position*, required more frequent tracheostomy relocation and were associated with longer time to decannulation.

Whether tracheostomy relocation should always be considered an unfavorable outcome remains debatable. Prior work from our institution supports that the use of DS reconstruction for complex LTS (with comorbidities or multilevel stenosis), in which a low tracheostomy (at the 5–6 or 6–7 ring level) preserves vascular supply above the stoma and maintains a safe distance from the reconstruction site, thereby reducing infection risk [3, 14]. Conversely, relocating a tracheostomy during reconstruction may create an additional site of tracheal injury, potentially compromising vascular supply and increasing infection risk in an already diseased airway. In pediatric populations (not specific to LTS), post‐tracheostomy complication rates range from 15% to 51%, with tracheostomy‐related stenosis reported in 1.7%–11.5% [3, 19, 20, 22, 23]. Prospective studies are needed to better distinguish between planned, strategy‐driven tracheostomy relocation and unplanned relocation performed during reconstruction, and to clarify their respective impact on outcomes.

### 4.2 Additional Considerations Regarding Confounding Factors

#### 4.2.1 Preoperative Airway Interventions

Preoperative airway interventions may act as confounding factors by inducing iatrogenic scarring. Such interventions may negatively impact prognosis, even in the presence of an optimally positioned tracheostomy, by increasing surgical complexity. These observations support the principle that, based on preoperative assessment, the first definitive airway intervention should be carefully selected, and that unnecessary intermediate procedures should be avoided.

#### 4.2.2 Lead Time to Reconstruction

The interval between tracheostomy placement and definitive airway reconstruction represents another important confounding factor. Reconstruction timing often depends on optimization of associated airway lesions and comorbidities, and this can delay the airway reconstruction. As a result, even an initially well‐positioned tracheostomy may induce secondary tracheal injury, including chronic inflammation, granulation tissue formation and cartilage weakening [1].

### 4.3 Institutional Reconstructive Strategy

In high grade stenoses (advanced Grade III and Grade IV), unlike LTR, CTR aims to completely excise dense diseased subglottic cicatricial tissue [6, 15]. Residual diseased mucosa may contribute to recurrence of stenosis. CTR is technically more challenging than LTR, and the surgeon must be sufficiently trained.

DS procedures were favored in patients with severe, multilevel stenosis or significant comorbidities, consistent with our institutional reconstructive strategy. Nevertheless, 40% of suboptimal cases underwent SS‐CTR, likely reflecting an adaptive surgical approach aimed at excising the tracheostomy en bloc with the stenosis when relocation would further compromise healthy trachea.

Within the optimal group, 37% underwent SS‐CTR. Two patients required unusually long resections (6 and 7 rings), which may account for the slightly greater resection lengths observed in this group compared with the nonoptimal group. These findings should be interpreted cautiously given subgroup heterogeneity and the retrospective design.

### 4.4 Tracheostomy Location Recommendations

Previous institutional reports underscored the importance of tracheostomy site selection, although without quantitative evidence [3, 14].

The proposed recommendations should be regarded as a pragmatic framework rather than a universal guideline. For reasons mentioned above, we prefer CTR for high‐grade stenosis; therefore, extrapolation of these findings to centers that favor LTR for these grades should be undertaken with caution.

**FIGURE 4:**
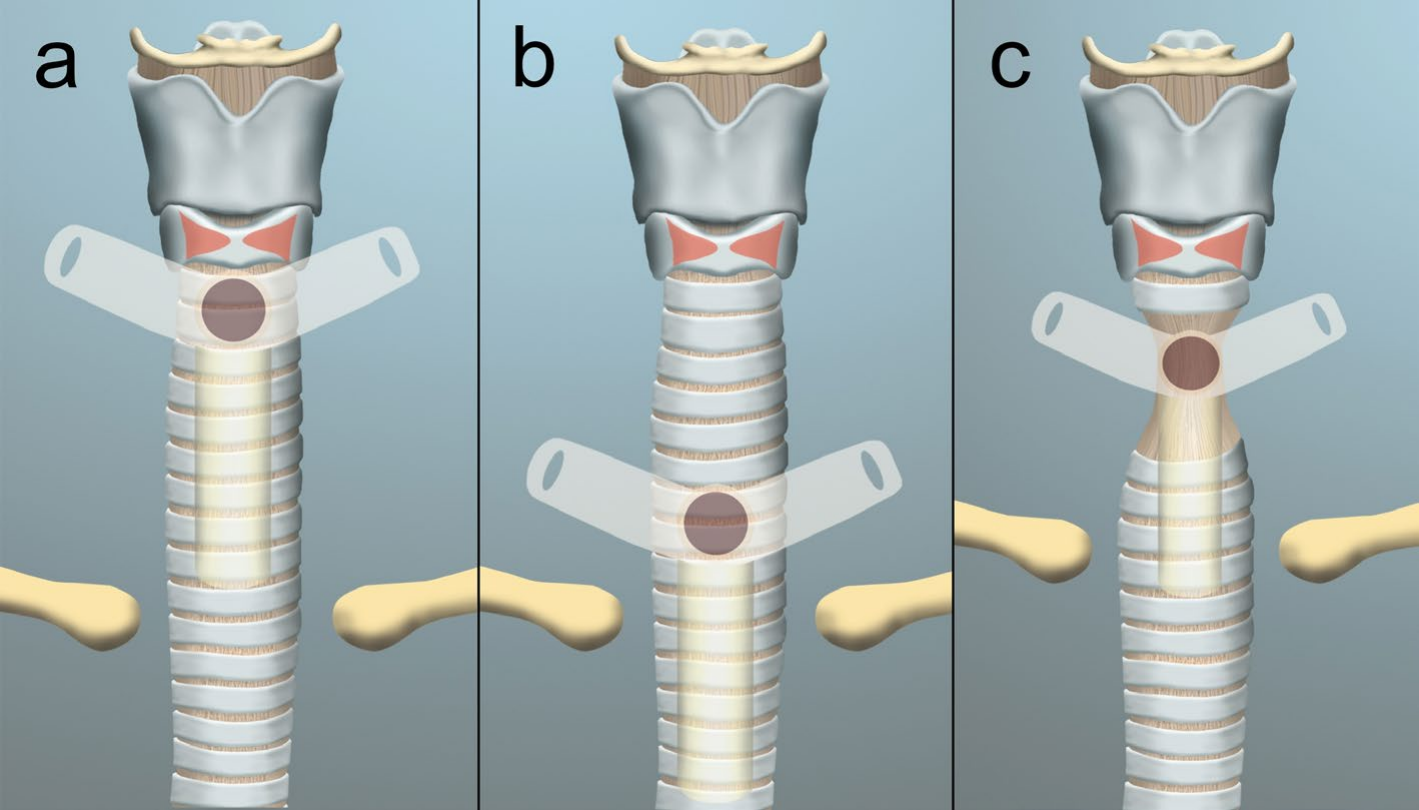
Recommended tracheostomy location according to stenosis location and type. (a) High tracheostomy for any grade of subglottic stenosis, in emergency setting without prior endoscopy, or when a low tracheostomy would be optimal but long surgical delay is expected, in order to avoid long‐term tracheal injury and allow subsequent relocation during airway reconstruction. (b) Low or very low tracheostomy for multilevel or trans‐glottic disease, significant comorbidities, or high‐grade glottic stenosis or webs. (c) Tracheostomy performed through the stenotic segment for cervical tracheal stenosis.

Based on our findings and institutional experience, we propose the following pragmatic considerations (see Figure 4a–c):

- *Isolated SGS without comorbidities (SS likely)*: a *high tracheostomy* facilitates limited resection or reconstruction (Figure 4a).
- *Multilevel or trans‐glottic disease, or significant comorbidities (DS likely)*: a *low or very low tracheostomy* to maintain well‐vascularized trachea and ensure a safe anastomosis [3, 14, 18] (Figure 4b).
- *High‐grade GS or high‐grade webs (DS likely)*: a *low or very low tracheostomy* minimizes infection risk, particularly of cartilage grafts [24, 25] (Figure 4b).
- *Cervical TS*: placing the stoma through the stenotic segment allows en bloc resection during segmental tracheal resection and anastomosis (Figure 4c).
- *Emergency cases without prior endoscopy*: a *high tracheostomy* is safest initially; facilitating SS procedures and preserving distal healthy trachea; if DS reconstruction is later required, a *low or very low tracheostomy* can be performed either as a secondary procedure or at the time of definitive surgery (Figure 4a).
- *Expected long delay to reconstruction*: when a *low or very low tracheostomy* would otherwise be optimal, an initially *high* tracheostomy that can later be relocated may reduce long‐term tracheal injury (Figure 4a).

## 5 Conclusions

Despite its limitations, this study suggests that tracheostomy location influences suitability for SS or DS airway reconstruction in pediatric LTS and may affect operative complexity and outcomes. Misplacement may increase healthy trachea resection length, necessitate stoma relocation, complicate reconstruction, and contribute to secondary stenosis. Whenever possible—even in urgent settings—endoscopic evaluation should precede tracheostomy to assess the stenosis level and severity. When clinically appropriate, avoidance of a *classic* position tracheostomy may help preserve healthy trachea and optimize future reconstructive options. Prospective studies are needed to validate this classification framework across centers with different reconstructive strategies.

## Funding

The authors have nothing to report.

## Conflicts of Interest

The authors declare no conflicts of interest.

## Supporting information


**Table S1:** Conceptual framework for tracheostomy position classification.

**Table S2:** Tracheal rings resected and time to decannulation in DS operations across groups.

## Data Availability

The data that support the findings of this study are available on request from the corresponding author. The data are not publicly available due to privacy or ethical restrictions.
