# Supplementary material for: Influence of Tracheostomy Level on Surgical Management of Pediatric Airway Stenosis
Source: Laryngoscope. 2026 Apr 15;136(9):3907–17. doi: 10.1002/lary.70569 (PMC13460537; doi:10.1002/lary.70569)
Supplement: Supplementary file 1 [file LARY-136-3907-s001.docx]

| Supplementary Table 1.  Conceptual framework for tracheostomy position classification | | | | |
| --- | --- | --- | --- | --- |
| **Classification** | **Stenosis characteristics** | **Comorbidity** | **Recommended tracheostomy level** | **Observed tracheostomy level** |
| Optimal | Isolated SGS | No | High | A |
|  |  | Yes | Low/very low | A |
|  | Glottic stenosis | No | High | A |
|  |  | Yes | Low/very low | A |
|  | Multilevel LTS | Yes or no | Low/very low | A |
|  | Tracheal stenosis | Yes or no | Through stenotic segment | A |
| Suboptimal | Any LTS | Yes or no | High/Classic/Low/very low | P |
| Nonoptimal | Any LTS | Yes or no | Away from recommendation (e.g. high vs low/very low) | D |
| *A: In accordance with expert recommendations; P: Partial discordance; D: Clear discordance* | | | | |
