# Supplementary material for: Influence of Tracheostomy Level on Surgical Management of Pediatric Airway Stenosis
Source: Laryngoscope. 2026 Apr 15;136(9):3907–17. doi: 10.1002/lary.70569 (PMC13460537; doi:10.1002/lary.70569)
Supplement: Supplementary file 2 [file LARY-136-3907-s002.docx]

| Supplementary Table 2.  Tracheal rings resected and time to decannulation in DS operations across groups | | | | | |
| --- | --- | --- | --- | --- | --- |
| **Variables** | **Median (IQR)** | **Median (IQR)** | **Median**  **Difference^b^** | **95% CI^b^** | **p-value^c^** |
| **Tracheal rings resected^a^** |  |  |  |  |  |
| Suboptimal vs Optimal | 5 (4-5) | 4 (3-4) | -1 | -2-0 | 0.027 |
| Suboptimal vs Nonoptimal | 5 (4-5) | 3 (2.5-4) | -1 | -2--1 | 0.004 |
| Optimal vs Nonoptimal | 4 (3-4) | 3 (2.5-4) | 0 | -1-1 | 0.548 |
|  |  |  |  |  |  |
| **Time to decannulation in DS operations (month)** |  |  |  |  |  |
| Suboptimal vs Optimal | 4 (2.5-8.6) | 3.8 (2-4.9) | -1.03 | -3.63-0.9 | 0.319 |
| Nonoptimal vs Suboptimal | 11.7 (4.6-19.8) | 4 (2.5-8.6) | 4.1 | -0.87-14.27 | 0.032 |
| Nonoptimal vs Optimal | 11.7 (4.6-19.8) | 3.8 (2-4.9) | 6.77 | 0.37-17.2 | 0.027 |
| *^a.^ CTR analyses include only patients with ≥1 tracheal ring resected (n=71). Seven patients with partial cricoid resection only were excluded*  *^b.^ Performed with Hodges-Lehman median difference with 95% confidence interval (median difference is calculated as left column - right column).*  *^c.^ Performed with Mann-Whitney U test* | | | | | |
